# Health state utility values by cancer stage: a systematic literature review

**DOI:** 10.1007/s10198-021-01335-8

**Published:** 2021-06-14

**Authors:** Mir-Masoud Pourrahmat, Ashley Kim, Anuraag R. Kansal, Marg Hux, Divya Pushkarna, Mir Sohail Fazeli, Karen C. Chung

**Affiliations:** 1 Evidinno Outcomes Research Inc., Vancouver, Canada; 2 GRAIL, Inc., Menlo Park, CA USA

**Keywords:** Health state utility, Disutility, Cancer, Cancer stage, Breast cancer, Lung cancer, Colorectal cancer, Cervical cancer, Systematic literature review, I10, I00

## Abstract

**Objectives:**

Cancer diagnoses at later stages are associated with a decrease in health-related quality of life (HRQOL). Health state utility values (HSUVs) reflect preference-based HRQOL and can vary based on cancer type, stage, treatment, and disease progression. Detecting and treating cancer at earlier stages may lead to improved HRQOL, which is important for value assessments. We describe published HSUVs by cancer type and stage.

**Methods:**

A systematic review was conducted using Embase, MEDLINE^®^, EconLit, and gray literature to identify studies published from January 1999 to September 2019 that reported HSUVs by cancer type and stage. Disutility values were calculated from differences in reported HSUVs across cancer stages.

**Results:**

From 13,872 publications, 27 were eligible for evidence synthesis. The most frequent cancer types were breast (*n* = 9), lung (*n* = 5), colorectal (*n* = 4), and cervical cancer (*n* = 3). Mean HSUVs decreased with increased cancer stage, with consistently lower values seen in stage IV or later-stage cancer across studies (e.g., − 0.74, − 0.44, and − 0.51 for breast, colorectal, and cervical cancer, respectively). Disutility values were highest between later-stage (metastatic or stage IV) cancers compared to earlier-stage (localized or stage I–III) cancers.

**Conclusions:**

This study provides a summary of HSUVs across different cancer types and stages that can inform economic evaluations. Despite the large variation in HSUVs overall, a consistent decline in HSUVs can be seen in the later stages, including stage IV. These findings indicate substantial impairment on individuals’ quality of life and suggest value in early detection and intervention.

**Supplementary Information:**

The online version contains supplementary material available at 10.1007/s10198-021-01335-8.

## Introduction

Cancer is the second leading cause of death globally and led to an estimated 9.6 million deaths in 2018 [1, 2]. Globally, approximately 1 in 6 deaths is due to cancer [2]. The economic cost of treating patients with cancer is also substantial, as the total cost of cancer care is estimated to reach more than $200 billion in 2020 in the US alone [3].

Cancer is a heterogeneous disease which can affect different parts of the body and can then spread to other organs. Cancer staging is used to help assess prognosis and inform treatment decisions [4]. For each cancer type, tumor size, lymph node involvement, and presence or absence of metastasis are used to define the stage of cancer, with stage 0 indicating tumors that have not spread to other sites (i.e., in situ), stages I–III indicating localized tumors ranging in size and lymph node involvement, and stage IV indicating distant metastasis [4].

Symptoms caused by cancer will vary depending on type, location, and stage, as well as the extent of involvement. Morbidity, which is typically increased with later-stage cancer diagnosis and disease progression, may be associated with widespread effects such as impairments in organ function, circulation, and other physiological processes [5]. General cancer-related morbidities such as fatigue, fever, and weight loss are often accompanied by cancer type-specific morbidities [6, 7].

In addition to disease-related morbidity, cancer treatment (i.e., standard antineoplastic therapy) has been associated with serious adverse events, including immunosuppression and infections. Additionally, recent cancer immunotherapy approaches, which are generally indicated in the treatment of advanced stage cancers, have been associated with immune-related adverse events (e.g., cytokine release syndrome) and central nervous system toxicities (e.g., encephalopathy, seizures) which can also impact patient quality of life [8–10].

The health-related burden of cancer on patients can be measured in terms of health-related quality of life (HRQOL), a multi-dimensional concept that includes domains related to physical, mental, emotional, and social functioning and focuses on the impact health status has on quality of life [11]. For patients with cancer, HRQOL can be impacted by factors such as fear, cancer-related fatigue, type of treatment received, social changes, and financial stress, as well as patient characteristics such as age, gender, and cancer type or site [12]. HRQOL can vary based on stage of cancer, disease progression, and cancer treatment/management [13]. Understanding how HRQOL is impacted by cancer types and stages is an essential part of fully describing the burden cancer places on patients.

Health state utility values (HSUV), a measure of preference-based HRQOL, represents individual's preference for being in a particular health state and are anchored on a 0 (dead) to 1 (full health) scale, with negative values representing health states worse than death [14]. Assessment of health state utilities can be either direct or indirect, where direct utility assessment involves mapping preferences directly onto the utility scale (e.g., standard gamble, time trade-off), and indirect utility assessment involves mapping preferences onto a utility scale via a generic HRQOL questionnaire (e.g., EQ-5D, short form six dimensions [SF-6D]) [14, 15]. Additionally, direct utility methods can include valuations of a patient’s own health state or valuations of health state vignettes, the latter of which may be valued by either patients who have experience with the relevant disease, by members of the general population who have no relevant clinical experience, or by health professionals or experts [14, 16, 17].

Disutilities, or decrease in utilities, can be used to assess HSUV reductions associated with adverse, disease-related outcomes such as progression and symptomatology, and adverse effects from chemotherapy and other treatments [14–16]. As part of the measure of benefit, utilities often have a strong influence on the results of cost-utility analyses, which are used in many countries to determine whether the cost of an intervention can be justified in terms of the health benefits it delivers [18–21]. Identifying gaps in our understanding of HSUVs and the potential clinical and economic impact may help to inform future research efforts.

Advances in cancer management include technologies that enable the earlier diagnosis of cancer (including potentially shifting diagnosis to earlier stages). As improved noninvasive screening techniques are developed that can simultaneously detect multiple cancer types years earlier than conventional methods [22–24], the economic value of these approaches must be assessed. Therefore, the objective of this investigation was to review and synthesize published estimates of HSUVs by cancer type and stage.

## Methods

### Study design and search strategy

A systematic literature search was performed using MEDLINE^®^ and Embase (via OvidSP), EconLit (via EBSCOhost), and proceedings from the Professional Society for Health Economics and Outcomes Research (ISPOR) 2017–2019 (including ISPOR Europe), International Conference on Health Economics (ICHE) 2017–2019, and International Society for Quality of Life Research (ISOQOL) 2017–2019. Study identification and eligibility criteria were developed using the evidence-based Population, Intervention, Comparator, and Outcome (PICO) framework as described by the Cochrane Collaboration’s handbook for Systematic Reviews of Interventions [25] (Online Resource 1).

Predefined search strategies were employed to identify articles reporting studies in any country that evaluated any cancer-related HSUVs stratified by cancer stage in adults, regardless of method of utility elicitation. We included clinical trials, observational studies, and surveys or data collection studies, and excluded case reports, pre-clinical studies, and economic analyses. Studies that recruited children/adolescents or patients with precancerous conditions were also excluded. Articles were limited to those in English published between January 1999 and September 9, 2019 (Online Resource 2). The selection of search terms to identify studies reporting HSUVs was informed by the list of recommended terms provided by the Canadian Agency for Drugs and Technologies in Health (CADTH) [26].

Titles and abstracts of initial search results were screened for eligibility by two analysts independently, with a third senior reviewer available to resolve discrepancies through arbitration. Abstracts eligible for inclusion were advanced to full-text screening, which was also performed by two independent reviewers.

### Data extraction and analysis

Data from eligible publications were extracted by two independent reviewers, organized into fields, and entered into Microsoft^®^ Excel spreadsheets for qualitative evidence synthesis. Whenever available, collected study data consisted of the study name, year, authors, country/location, health state, utility assessment or development method, sample source, and patient eligibility criteria. Patient characteristics of interest included the population definition, age, sex, cancer type, cancer stage, duration of disease, and treatment status.

HSUV estimates were also extracted by cancer stage and type, when available. HSUVs were reported by numerical tumor/lymph/metastasis (TNM) cancer stage if available, or classified as early (i.e., localized and/or defined chronologically) or later (typically distant spread or metastasis). To investigate how progression through cancer stages affects HSUVs, differences in HSUV between stages/states were calculated to produce disutility values.

Results are presented as mean or median and SD or 95% CI as indicated.

### Quality of reporting

A quality assessment of study design, data collection techniques, and analysis and interpretation of results was performed by two independent methodologists using elements of critical appraisal from the checklist in the National Institute for Health and Care Excellence (NICE) single technology appraisal template for economic evaluations [27].

## Results

### Study selection

**Fig. 1:**
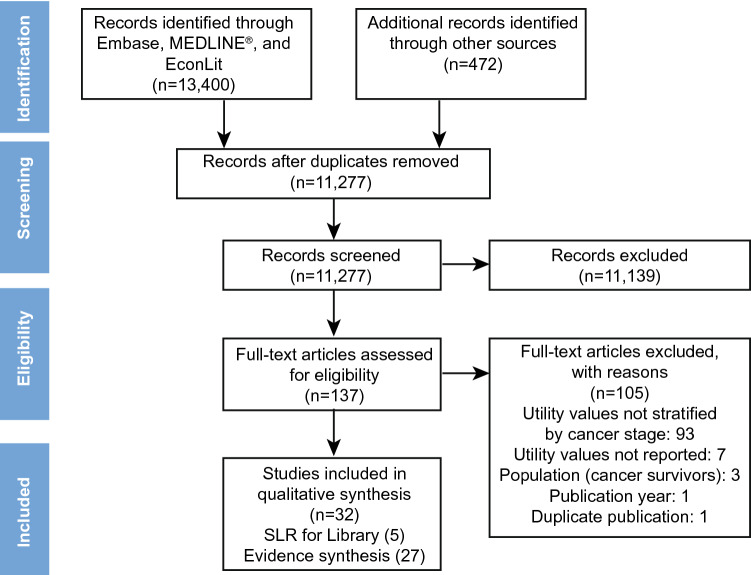
PRISMA diagram. Only articles in English and published between January 1999 and September 9, 2019 were included. Studies with children/adolescents or patients with precancerous conditions were not included. *SLR* systematic literature review

A total of 13,872 records initially identified by the database search underwent title and abstract screening. Of these, 137 citations were included for full-text screenings, from which 27 studies were eligible for inclusion in qualitative evidence synthesis. A Preferred Reporting Items for Systematic Reviews and Meta-Analyses (PRISMA) flow diagram illustrating the study selection process is presented in Fig. 1.

### Study characteristics

Full study characteristics are summarized in Online Resource 3. Of the 27 studies analyzed, 25 were cross-sectional and two were prospective observational studies. Studies were most commonly performed in multiple countries (*n* = 4), China (*n* = 4), South Korea (*n* = 3), the United States (*n* = 3), or Japan (*n* = 2), while the remaining studies took place in individual countries in Europe (*n* = 6), the Asia–Pacific region (*n* = 3), or the Americas (*n* = 2).

HSUVs were elicited by indirect methods (n = 16), direct methods (n = 7), or a combination of both methods (n = 4).

## Patient characteristics

The mean (SD) patient age across 16 studies with available data was 55.7 (11.3) years, and 32.2% of patients (based on 23 studies) were male. From the five studies that reported race, the mean proportion of Caucasians was 73.1% (median [range]: 80.2% [46.9–93.8%]). The mean (SD) duration of disease was reported in only two studies: 232.4 (230.8) days in patients with advanced non-small cell lung cancer (NSCLC) [28] and 7.8 (4.6) years in patients with end-stage breast, prostate, or colorectal cancer [29]. Patient characteristics are summarized by study in Online Resource 3.

### Reported outcomes

**Fig. 2:**
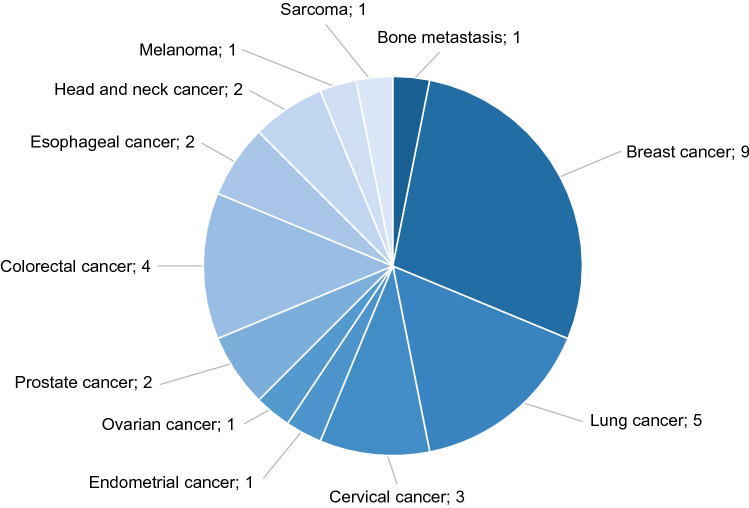
Numbers of studies by cancer type based on publications included in the systematic review

Fifteen studies reported HSUVs by cancer stages, while 12 studies reported HSUVs by cancer health states, from which ‘earlier’ or ‘later’ stage cancers were derived. Breast cancer was the most reported type (*n* = 9 studies), followed by lung (*n* = 5), colorectal (CRC; *n* = 4), and cervical cancer (*n* = 3). Prostate, esophageal, and head and neck cancer were reported in two studies each (Fig. 2). All HSUVs reported are summarized in Online Resource 4. HSUVs for bone cancer [16], endometrial cancer [30], and sarcoma [31] were each reported in single studies and are reported in Online Resource 4 only.

### Breast cancer

A total of nine studies reported HSUVs in breast cancer: six that reported on patients with the condition [29, 30, 32–35], two on individuals from the general population (*n* = 2) [36, 37], and one on healthcare experts [38]. HSUVs were based on direct methods in three studies [36–38], indirect methods in five studies [29, 30, 32, 33, 35], or both in one study [34].

Mean HSUVs ranged across all studies from 0.56 to 0.90 in stage I, 0.48–0.79 in stage II, 0.45–0.77 in stage III, and 0.35–0.86 in stage IV.

**Fig. 3:**
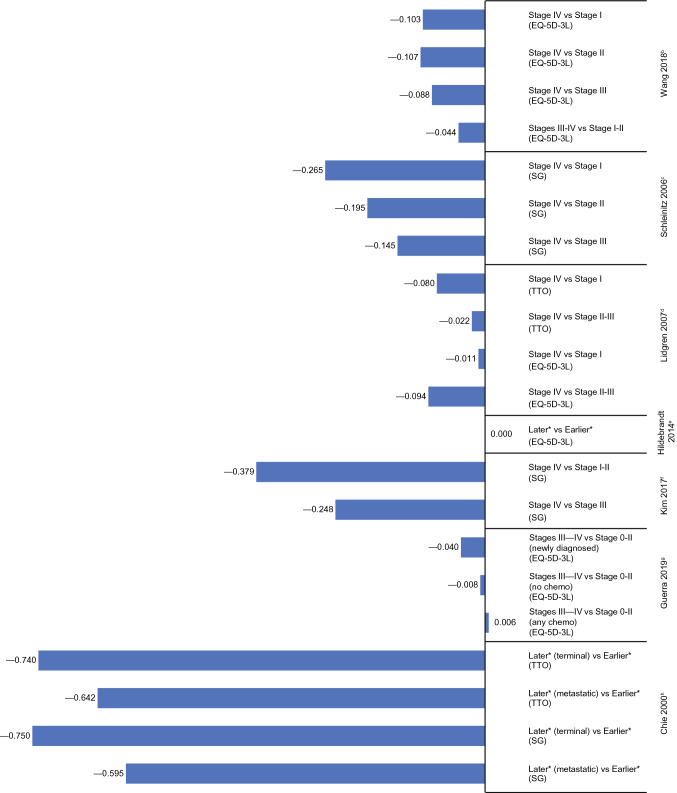
Disutility values calculated by stage of breast cancer^a^. *SG* standard gamble, *TTO* time trade-off. ^a^Disutility values presented above are calculated for each stage as change in utility from previous stage(s). ^b^Wang [33]: EQ-5D-3L, own health state, patients. ^c^Schleinitz [37]: SG, hypothetical health states, general population (females). ^d^Lidgren [34]: EQ-5D-3L, own health state, patients; TTO: hypothetical health states, patients. ^e^Hildebrandt [30]: EQ-5D-3L, own health state, patients and healthy controls. ^f^Kim [36]: SG, hypothetical health states, general population. ^g^Guerra [35]: EQ-5D-3L, own health state, patients. ^h^Chie [38]: SG and TTO, hypothetical health states, healthcare experts. *Health states that signified localized cancers were labeled as “earlier” stage and distant or metastasized as “later” stage

Of the nine studies on breast cancer, six reported lower HSUVs for distant/metastatic disease compared with local disease, producing negative disutilities (Fig. 3). The first two studies used indirect methods (EQ-5D-3L) to elicit HSUVs. In the first study, Guerra et al. [35] assessed patients with newly diagnosed breast cancer who had not initiated routine treatment. The calculated disutility at stage III/IV vs stage 0–II was –0.040 based on HSUVs of 0.689 and 0.729, respectively. Corresponding disutilities were − 0.008 and + 0.006 for patients who had initiated treatment without or with chemotherapy, respectively. In the second study, which was performed with patients from a cancer screening program in China, Wang et al. [33] reported HSUVs that were significantly decreased with increased stage (0.789, 0.793, 0.774, and 0.686 in stages I, II, III, and IV, respectively; *P* < 0.001), producing disutility values of –0.103 for stage IV vs I and − 0.088 for stage IV vs III (Fig. 3).

Three studies that used direct methods also reported lower HSUVs in the later stages of breast cancer. Using the standard gamble (SG) approach, Kim et al. [36] reported HSUVs of 0.731, 0.600, and 0.352 for stages I–II, III, and IV, respectively, generating higher disutility values for stage I–II vs IV (–0.38) than stage III vs IV (− 0.25). In a separate study by Schleinitz et al. [37], women aged ≥ 25 years in a convenience sample from US primary care clinics and the community were assessed via SG, revealing that the highest disutility was produced for stage IV vs I (− 0.265), compared with stage IV vs II (− 0.195) or stage IV vs III (− 0.142). This trend was consistent among subgroups based on ethnicity, age, education level, household income, marital status, and family history of breast cancer [37]. In the third study, Chie et al. [38] assessed HSUVs via an expert panel (*n* = 31), revealing higher disutilities for later (metastatic) vs earlier (localized) disease using both SG (− 0.750) and time trade-off (TTO; − 0.642) approaches (Fig. 3). In the final study that reported lower HSUVs in later-stage breast cancer, Lidgren et al. [34] used both direct (TTO) and indirect (EQ-5D-3L) methods in patients who received chemotherapy and hormone therapy. Based on TTO, disutilities were higher for stage IV vs I (− 0.080) than stage IV vs II–III (− 0.022); however, HSUVs based on the EQ-5D-3L resulted in a lower disutility for stage IV vs I (− 0.011) than stage IV vs II–III (− 0.094) (Fig. 3).

For the remaining three breast cancer studies, Hildebrandt et al. [30] reported no difference in median HSUVs between earlier (primary, nonmetastatic) and later (metastatic) disease (both 0.887 based on EQ-5D-3L) (Fig. 3); while Wood et al. [32] reported HSUVs for stage III–IV only (ranged from 0.62 to 0.78), and Farkkila et al. [29] only reported HSUVs for end-stage disease (0.447 based on EQ-5D-3L and 0.718 based on 15D) (see Online Resource 4).

### Colorectal cancer

HSUVs for CRC were reported in four studies: three in CRC patients [29, 39, 40], and one in individuals from the general Korean population [41]. HSUVs were reported based on direct methods (*n* = 1) [41], indirect methods (*n* = 2) [29, 39], or both (*n* = 1) [40]. Overall, mean HSUV ranges including subgroups were 0.64–0.77 for stage I, 0.56–0.72 for stage II–III, and 0.3–0.53 for stage IV.

**Fig. 4:**
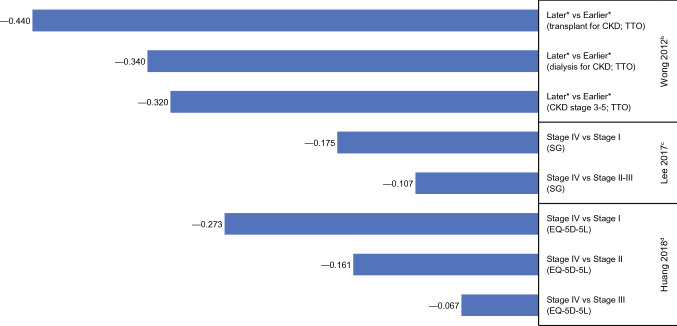
Disutility values calculated by stage of colorectal cancer^a^. *CKD* chronic kidney disease, *SG* standard gamble, *TTO* time trade-off. ^a^Disutility values presented above are calculated for each stage as change in utility from previous stage(s). ^b^Wong [40]: TTO, hypothetical health states, patients with chronic kidney disease. ^c^Lee [41]: SG, hypothetical health states, general population. ^d^Huang [39]: EQ-5D-5L, own health state, patients. *Health states that signified localized cancers were labeled as “earlier” stage and distant or metastasized as “later” stage

Of the four studies, three reported HSUV at multiple stages, all of which showed lower HSUVs with more advanced disease. Calculated disutility values from these studies are presented in Fig. 4. In a study by Huang et al. [39], HSUVs generated via EQ-5D-5L in patients who received treatment for newly diagnosed CRC yielded higher disutility values for stage IV vs I (− 0.273), compared with stage IV vs II (− 0.161) and stage IV vs III (− 0.07). In a study by Wong et al. [40], hypothetical CRC in patients with stage 3–5 chronic kidney disease (CKD) assessed via TTO produced a disutility of − 0.320 for later (metastatic) vs earlier (localized) disease. Corresponding disutility values were − 0.340 and − 0.440 from patients who were undergoing dialysis or kidney transplant, respectively. Lee et al. [41] used SG in individuals from the general population, revealing greater disutilities for stage IV vs I (− 0.175) than stage IV vs II–III (− 0.107), with consistent findings among subgroups based on gender, age, education level, occupation, monthly income, hospitalization, and morbidity. Farkkila et al. [29] only reported an HSUV for stage IV CRC (0.662 based on the EQ-5D-3L and 0.764 based on the 15D), which is summarized in Online Resource 4.

### Lung cancer

Five studies assessed HSUVs in lung cancer from patients with the disease or individuals without cancer. Lung cancer subtypes comprised one study in squamous cell lung cancer [42], three studies in NSCLC [28, 43, 44], and one study in general lung cancer (*n* = 1) [45]. Four studies reported HSUVs derived from indirect methods [28, 43–45], and one from direct methods [42]. Studies by Wolff et al. [44] and Iyer et al. [28] that only reported HSUVs for early disease or late disease are summarized in Online Resource 4. Across all studies, mean utility scores ranged from 0.59 to 0.86 in stage I, 0.56–0.81 in stage II, 0.27–0.89 in stage III, and 0.66–0.84 in stage IV.

**Fig. 5:**
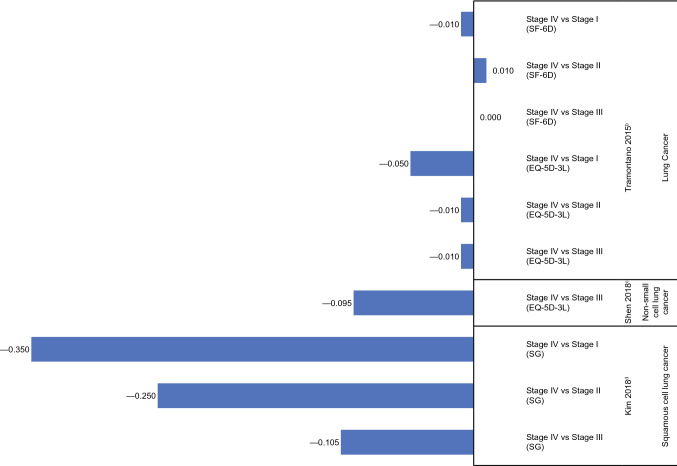
Disutility values calculated by stage of lung cancer^a^. *SG* standard gamble, *SF-6D* Short-Form 6 Dimension. ^a^Disutility values presented above are calculated for each stage as change in utility from previous stage(s). ^b^Tramontano [45]: EQ-5D-3L and SF-6D, own health state, patients. ^c^Shen [43]: EQ-5D-3L, own health state, patients. ^d^Kim [36]: SG, hypothetical health states, general population

Disutility values for the three studies in newly diagnosed lung cancer across multiple stages are presented in Fig. 5. In a study by Tramontano et al. [45], HSUVs based on the EQ-5D-3L at diagnosis produced a larger disutility for stage IV vs I (− 0.050) than stage IV vs III (− 0.010) or IV vs II (also − 0.010). In the same patients, disutilities calculated based on the SF-6D (which was derived from the SF-12v2) were smaller overall, but still largest for stage IV vs I (− 0.050) compared with stage IV vs III (− 0.010) or stage IV vs II (− 0.020). A reassessment of these patients with the EQ-5D-3L at 11–13 months post diagnosis revealed a disutility of − 0.05 for stage IV vs I. While most subgroup analyses in this study showed a consistent trend of decreasing HSUVs with later stages, treatment-based subgroups defined as patients receiving chemotherapy, platinum surgery with chemotherapy, and platinum chemotherapy showed a trend for slight increases in HSUVs at stage IV vs stage III [45]. In a separate study by Shen et al. [43], HSUVs (based on EQ-5D-3L) in patients with NSCLC resulted in a disutility of − 0.095 for stage IV vs III. In the third study, Kim et al. [42] used SG to elicit HSUVs in adults from the general population in Korea, revealing that the largest disutility occurred for stage IV vs I (− 0.350) compared with stage IV vs II (− 0.250) and stage IV vs III (− 0.105). Consistent results were observed in this study among subgroups based on gender, age, education level, occupation, monthly income, hospitalization, and morbidity.

### Cervical cancer

Three studies reported HSUVs for multiple stages of cervical cancer; one study each in patients with the disease [46], individuals without cancer [47], or both [30]. Two studies generated HSUVs using only indirect methods [30, 46] and one study used direct methods [47]. Mean utility scores across studies ranged from 0.63 to 0.85 in stage I, 0.50–0.76 in stage II, 0.52–0.71 in stage III, and 0.18–0.77 in stage IV.

**Fig. 6:**
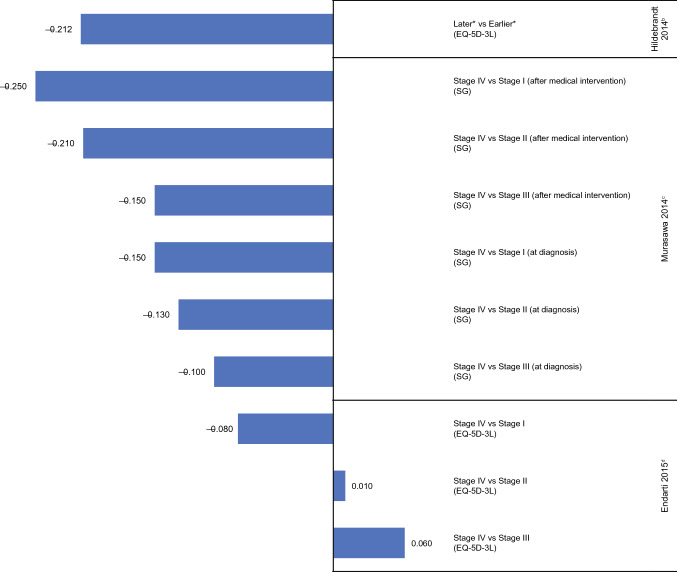
Disutility values calculated from HSUV reported in patients with cervical cancer^a^. *SG* standard gamble. ^a^Disutility values presented above are calculated for each stage as change in utility from previous stage(s). ^b^Hildebrandt [30]: EQ-5D-3L, own health state, patients and healthy controls. ^c^Murasawa [47]: SG, hypothetical health states, general population (female students). ^d^Endarti [46]: EQ-5D-3L, own health state, patients. *Health states that signified localized cancers were labeled as “earlier” stage and distant or metastasized as “later” stage

All studies showed increased disutility with advancing disease (Fig. 6). When Endarti et al. [46] examined patients with cervical cancer, disutilities based on EQ-5D-3L were greatest for stage IV vs stage I (− 0.080) compared with stage IV vs II (+ 0.010) and stage IV vs III (+ 0.060). In the second study, Murasawa et al. [47] assessed HSUVs based on hypothetical cervical cancer at diagnosis and after medical intervention using SG in healthy female students at a nursing university. Disutilities calculated for stage IV vs stage I were the largest at both diagnosis (− 0.150) and after medical intervention (− 0.250) compared with stage IV vs II (− 0.130 and − 0.250, respectively) or stage IV vs III (− 0.100 and − 0.150, respectively) (Fig. 6). In the final study, Hildebrandt et al. [30] elicited HSUVs in patients with cervical cancer using the EQ-5D-3L, which produced a disutility of − 0.212 for later (advanced) vs earlier (primary) disease based on HSUVs of 0.788 and 1.000, respectively.

### Prostate cancer

Two studies reported HSUVs in patients with prostate cancer [29, 47], both of which used indirect methods. Mean HSUV ranged from 0.87 in earlier (localized) disease to 0.55 in later (end-stage) disease. In the first study, Murasawa et al. [48] used the EQ-5D-5L to generate HSUVs at multiple stages, revealing a decrease from 0.845 in later (distant metastatic) disease vs 0.865 in earlier (localized with or without progression) disease to produce a disutility of –0.020. In the other study (Farkkila et al. [29]) a disutility value was not calculated as HSUVs were reported for end-stage disease only of 0.551 and 0.694 based on the EQ-5D-3L and 15D, respectively.

### Head and neck cancer

**Fig. 7:**
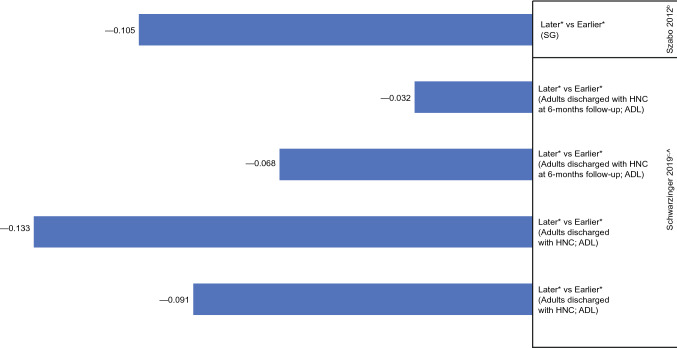
Disutility values calculated by stage of head and neck cancer^a^. *ADL* activities of daily living, *HNC* head and neck cancer, *SG* standard gamble. ^a^Disutility values presented above are calculated for each stage as change in utility from previous stage(s). ^b^Szabo [50]: SG, hypothetical health states, general population. ^c^Schwarzinger [49]: ADL, own health state, patients. ^^^A two-parameter graded response model (Item Response Theory) was estimated from all ADLs and the latent health state scale underlying ADLs was calibrated with the French EQ-5D-3L social value set. *Health states that signified localized cancers were labeled as “earlier” stage and distant or metastasized as “later” stage.

Two studies estimated HSUVs for head and neck cancer in multiple cancer stages [49, 50], which ranged from 0.316 to 0.699 in early (nonmetastatic) disease and 0.269–0.647 in later (metastatic) disease, resulting in greater disutility at later vs earlier disease (Fig. 7).

In the first study, Schwarzinger et al. [49] determined HSUVs based on activities of daily living (ADL) assessments in adults with head and neck squamous cell carcinoma. A two-parameter graded response model was estimated from all ADLs, and the latent health state scale underlying ADLs was calibrated with the French EQ-5D-3L social value set. In the full study population, the calculated disutility for later (metastatic) vs earlier (locally advanced) stages was − 0.091. When patients were stratified by age at diagnosis and number of comorbidities, those diagnosed at age 75–79 years reported the largest disutility for later vs earlier stages (− 0.133). In the second study, Szabo et al. [50] used SG to generate HSUVs from members of the Canadian general public resulting in a disutility of –0.105 for later (metastatic) vs earlier (locoregional) disease.

### Esophageal cancer

Two studies reported HSUVs in multiple stages of esophageal cancer. One study used direct and indirect methods to elicit utilities from patients or healthy controls [51], while the other used an indirect method in patients only [52]. The mean HSUVs across studies ranged from 0.60 to 0.82 in stage I, 0.46–0.81 in stage II, 0.15–0.80 in stage III, and were 0.66 in stage IV.

**Fig. 8:**
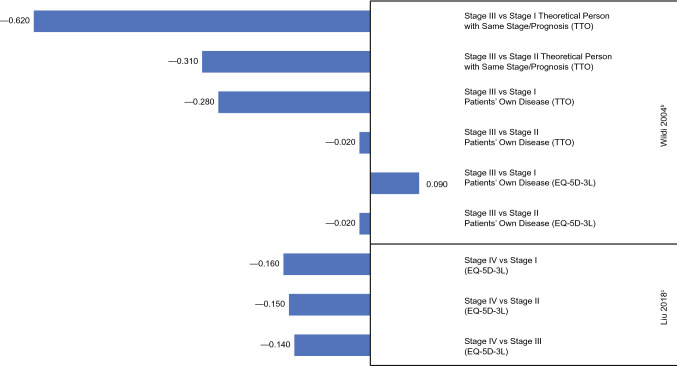
Disutility values calculated by stage of esophageal cancer^a^. TTO, time trade-off. ^a^Disutility values presented above are calculated for each stage as change in utility from previous stage(s). ^b^Wildi [51]: TTO, own health state and hypothetical health states, patients. ^c^Liu [52]: EQ-5D-3L, own health state, patients and healthy controls

Disutilities from these studies are summarized in Fig. 8. In the first study, Wildi et al. [51] elicited HSUVs from patients with a pathologically confirmed, newly diagnosed adenocarcinoma, or squamous cell carcinoma of the esophagus based on both the patients’ own disease and the disease of a theoretical person. Cancer stages were based on Surveillance, Epidemiology, and End Results Program (SEER) summary staging definitions [53]: stage I was defined as localized, stage II as regional, and stage III as metastatic disease. Based on TTO, the disutility for the theoretical person was greater for stage III vs I (− 0.620) compared with stage III vs II (− 0.310), while corresponding disutilities for the patient’s own disease were − 0.280 and − 0.020, respectively (Fig. 8). The disutilities derived using the EQ-5D-3L for the patient’s own disease were − 0.020 for stage III vs II and + 0.090 for stage III vs I [51]. Thus, patients consistently rated their own utility better than that of the theoretical person.

In the second study, Liu et al. [52] produced HSUVs based on the EQ-5D-3L in healthy controls and patients with esophageal cancer who had been discharged more than a year prior to taking the assessment. In these patients, disutilities were greatest for stage IV vs stage I (− 0.160), compared with stage IV vs II (− 0.150) and stage IV vs III (− 0.140). Notably, HSUVs for the patients were decreased compared with healthy controls by 0.289, 0.303, 0.296, and 0.505 at stages I, II, III, and IV, respectively (all *P* < 0.001).

### Melanoma

One study by Tromme et al. [54] assessed HSUVs using the EQ-5D-5L in patients with melanoma who were in remission or undergoing treatment. Given the short and varied duration of treatment approaches (especially surgery), utilities were assessed based on specific treatment durations (as assumed by experts) for each stage of cancer, while patients in remission were assessed based on different durations of remission. In patients undergoing treatment, disutility values were + 0.004 for stage IV vs stages I/II and + 0.048 for stage IV vs III. For patients in remission, corresponding disutility values were − 0.006 and + 0.093, respectively (data not shown).

### Study quality assessment

In the data quality assessment, a low overall risk was observed for the study design aspect, as the majority of studies indicated HSUV in the research question (Online Resource 5). However, some variation in risk was observed for data collection, as all studies reported methods used to value health states; however, two studies did not provide details of study subjects. Eleven studies reported the utility directly from the patient samples for relevant groups, whereas 16 studies used modeling such as regression analyses to estimate utility. Of the studies that used modeling, 11 studies did not provide justification for the choice of model used and the key parameters on which the model was based. The included studies had low risk of other sources of bias, as all provided an answer to the study question and 25 studies reported conclusions based on the results, while two studies reported generalized conclusions.

## Discussion

This review focuses on published studies reporting HSUVs, a measure of preference-based HRQOL, across multiple cancer types and stages, highlighting gaps in the evidence with regard to stage-specific health state utilities and disutilities. While the impact of cancer on individuals’ HRQOL is well documented [12, 13, 55], there is limited information regarding the HRQOL impact of cancer stage, specifically stage at diagnosis.

Previous systematic reviews and studies have generally reported HSUVs before and after cancer progression or by lines of chemotherapy and only within a single cancer type or stage [14, 56–58]. Our review assesses and compares HSUVs across various cancer types and stages, thereby identifying important differences in HSUVs based on cancer stage across cancer types. The largest disutilities were found between later-stage (metastatic or stage IV) cancers compared to earlier-stage (localized or stages I–III) cancers; though, even within the earlier stages, HSUVs tended to decline with increasing stage.

The studies reviewed the most commonly addressed HSUVs associated with higher-incidence cancers such as breast, colorectal, and lung cancers, all of which cover only about 40% of cancer incidence. Data on utility values for other cancer types, which represent more than 60% of incident cancers, is in fact, sparse [59]. Thus, while early detection of cancer is a critical element in improving cancer outcomes [60], our review suggests important gaps in the literature on HSUVs between stages within a cancer type, particularly for lower-incidence cancers.

We also found substantial heterogeneity in HSUVs across stages, particularly in later cancer stages. The potential cause of this variability is likely multifactorial, including the timing of assessment (e.g., at diagnosis vs during or after cancer treatment), utility elicitation method, types of responders (e.g., patients, healthy subjects, clinical experts), disease-specific factors, and variations with regard to health state descriptions.

While previous studies have assessed differences in HSUVs by elicitation method and type of responder valuing the health state [61], our review did not see a consistent bias with regards to method in the estimate of utility, though careful interpretation of values is warranted given the differences in methodologies and patient population inclusion. For instance, the TTO was used alongside the EQ-5D-3L in the studies by Lidgren et al. [34] and Wildi et al. [51] to obtain utility values in cancer patients. Clear differences in breast cancer health state utilities were observed when assessing utility values based on TTO compared to EQ-5D-3L in the Lidgren study. Mean TTO values were higher than mean EQ-5D-3L values for all cancer stages. Yet, the study by Wildi et al. did not find appreciable differences between the TTO and EQ-5D-3L across stages of esophageal cancer. Of the different HSUV derivation methods used in our review, the EQ-5D was most frequently used (*n* = 18), followed by direct elicitation methods (SG and TTO; *n* = 11), the SF-6D (*n* = 1), and the 15D (*n* = 1).

Despite differences in mean health utility values across studies among similar cancer types and stages, consistent declines in utility (i.e., disutility) were observed between earlier-stage (e.g., stage I) and later-stage (e.g., stage IV) cancers. Specifically, disutilities were notably highest between stage IV and stage I cancer, followed by stage IV and stage II cancer, and lowest between stage IV and stage III cancer, across all cancer types. This is likely due to the fact that earlier-stage (e.g., stages I–II) cancers tend to be associated with decreased symptom impact and can often be treated with curative intent, whereas later-stage or metastatic (e.g., stage IV) cancers are often associated with increased symptomatology due to tumor growth and/or spread to other organs and can require ongoing palliative treatment with chemotherapy [62]. In addition, the HSUVs reported in the study by Guerra et al. [35] were slightly lower in patients undergoing chemotherapy compared to any other cancer treatment, and even lower in patients undergoing sequential chemotherapy, irrespective of stage. This finding may be explained by an overload of distressing symptoms secondary to sequential cancer treatments or disease progression in those with advanced disease. Thus, if earlier detection of cancer could avoid a series of sequential treatments, patients may experience a higher quality of life without the toxic side effects and psychological burden.

While there may be several possible subject- and study-specific sources of variation in the reported HSUVs by cancer stage and type, our findings have shown differences in HRQOL across stages and increased disutility with later-stage cancer diagnosis. Further research is warranted to further inform HSUVs by cancer type and stage to enable accurate representation in cost-utility models.

## Strengths and limitations

A notable strength of this study is the exhaustive search strategy implemented to identify studies of interest. To our knowledge, this is the only study that provides HSUVs across cancer stages within multiple cancer types. The most notable limitation was the large heterogeneity across the evaluated studies. In addition to the large variation in the number of patients within each cancer stage, nearly half of the included studies had sample sizes of < 200, and some studies used a convenience-based sampling method for recruitment, which may limit the generalizability of the results. In several studies, HSUV estimates also varied by type of treatment received as well. Furthermore, in addition to fundamental differences across studies in how and from whom the utility values were collected, there was considerable variability in the classification of cancer stages, though we attempted to provide uniformity for comparison purposes, and categorized certain cancer health states that specified localization of cancer as “earlier”-stage cancer and distant metastases as “later”-stage cancer based on clinical guidelines. Finally, we only included English language publications, which may have excluded studies relevant to the research question.

## Conclusions

This systematic review provides health state utility estimates across cancer types and stages. Overall, this review demonstrates that cancers diagnosed at a later stage are associated with substantial HRQOL impairment, with considerable decline observed from stage I to stage IV. HSUVs are essential parameters in model-based economic evaluations that inform healthcare resource allocation and aid important policy decisions regarding the reimbursement of health technologies. These results could be informative for determining the value of novel approaches to early detection of cancer.

## Supplementary Information

Below is the link to the electronic supplementary material.

Supplementary file1 (DOCX 20 KB)

Supplementary file2 (DOCX 27 KB)

Supplementary file3 (DOCX 53 KB)

Supplementary file4 (XLSX 57 KB)

Supplementary file5 (DOCX 26 KB)

## Data Availability

Not applicable.

## References

[1] Bray F, Ferlay J, Soerjomataram I, Siegel RL, Torre LA, Jemal A (2018). Global cancer statistics 2018: GLOBOCAN estimates of incidence and mortality worldwide for 36 cancers in 185 countries. CA Cancer J. Clin..

[2] World Health Organization: Cancer. https://www.who.int/news-room/fact-sheets/detail/cancer. Accessed 22 Dec 2020

[3] Mariotto AB, Enewold L, Zhao J, Zeruto CA, Yabroff KR (2020). Medical care costs associated with cancer survivorship in the United States. Cancer Epidemiol. Biomark. Prev..

[4] Cancer Staging Guide. https://www.nccn.org/patients/resources/diagnosis/staging.aspx. Accessed 25 Sept 2020

[5] Signs and symptoms of cancer | do i have cancer?, https://www.cancer.org/cancer/cancer-basics/signs-and-symptoms-of-cancer.html. Accessed 26 Sept 2020

[6] Kurzrock R (2001). The role of cytokines in cancer-related fatigue. Cancer.

[7] Gegechkori N, Haines L, Lin JJ (2017). Long term and latent side effects of specific cancer types. Med. Clin. N. Am..

[8] Haanen JBG, Carbonnel F, Robert C, Kerr KM, Peters S, Larkin J, Jordan K (2017). Management of toxicities from immunotherapy: ESMO clinical practice guidelines for diagnosis, treatment and follow-up†. Ann. Oncol..

[9] Bonifant CL, Jackson HJ, Brentjens RJ, Curran KJ (2016). Toxicity and management in CAR T-cell therapy. Mol. Ther. Oncolytics..

[10] Carter, S., Thurston, D.E.: Immuno-oncology agents for cancer therapy. https://www.pharmaceutical-journal.com/research/review-article/immuno-oncology-agents-for-cancer-therapy/20207825.article. Accessed 22 Dec 2020

[11] Healthy People 2020 [Internet]. Washington, DC:U.S. Department of Health and Human Services, Office of Disease Prevention and Health Promotion, Washington, DC. https://www.healthypeople.gov/2020/topics-objectives/topic/health-related-quality-of-life-well-being. Accessed 22 Dec 2020

[12] Peters E, Mendoza Schulz L, Reuss-Borst M (2016). Quality of life after cancer—how the extent of impairment is influenced by patient characteristics. BMC Cancer.

[13] Shrestha A, Martin C, Burton M, Walters S, Collins K, Wyld L (2019). Quality of life versus length of life considerations in cancer patients: a systematic literature review. Psychooncology..

[14] Paracha N, Abdulla A, MacGilchrist KS (2018). Systematic review of health state utility values in metastatic non-small cell lung cancer with a focus on previously treated patients. Health Qual. Life Outcomes.

[15] Li L, Severens JL, Mandrik O (2019). Disutility associated with cancer screening programs: a systematic review. PLoS ONE.

[16] Matza LS, Chung K, Van Brunt K, Brazier JE, Braun A, Currie B, Palsgrove A, Davies E, Body JJ (2014). Health state utilities for skeletal-related events secondary to bone metastases. Eur J Health Econ..

[17] Wolowacz SE, Briggs A, Belozeroff V, Clarke P, Doward L, Goeree R (2016). Estimating health-state utility for economic models in clinical studies: an ISPOR good research practices task force report. Value Health..

[18] Canadian Agency for Drugs and Technologies in Health (2006). Guidelines for the economic evaluation of health technologies.

[19] Guide to the methods of technology appraisal 2013. https://www.nice.org.uk/guidance/pmg9/resources/guide-to-the-methods-of-technology-appraisal-2013-pdf-2007975843781. Accessed 26 Sept 2020

[20] Australian Government Department of Health.: Guidelines for preparing submissions to the Pharmaceutical Benefits Advisory Committee (PBAC). https://pbac.pbs.gov.au/. Accessed 26 Sept 2020

[21] Ara, R., Wailoo, A.: NICE DSU Technical Support Document 12: The Use of Health State Utility Values in Decision Models [Internet]. National Institute for Health and Care Excellence (NICE), London (2011) 28481493

[22] Aravanis AM, Lee M, Klausner RD (2017). Next-generation sequencing of circulating tumor DNA for early cancer detection. Cell.

[23] Chen X, Gole J, Gore A, He Q, Lu M, Min J, Yuan Z, Yang X, Jiang Y, Zhang T, Suo C, Li X, Cheng L, Zhang Z, Niu H, Li Z, Xie Z, Shi H, Zhang X, Fan M, Wang X, Yang Y, Dang J, McConnell C, Zhang J, Wang J, Yu S, Ye W, Gao Y, Zhang K, Liu R, Jin L (2020). Non-invasive early detection of cancer four years before conventional diagnosis using a blood test. Nat. Commun..

[24] Chabon JJ, Hamilton EG, Kurtz DM, Esfahani MS, Moding EJ, Stehr H, Schroers-Martin J, Nabet BY, Chen B, Chaudhuri AA, Liu CL, Hui AB, Jin MC, Azad TD, Almanza D, Jeon Y-J, Nesselbush MC, Keh LCT, Bonilla RF, Yoo CH, Ko RB, Chen EL, Merriott DJ, Massion PP, Mansfield AS, Jen J, Ren HZ, Lin SH, Costantino CL, Burr R, Tibshirani R, Gambhir SS, Berry GJ, Jensen KC, West RB, Neal JW, Wakelee HA, Loo BW, Kunder CA, Leung AN, Lui NS, Berry MF, Shrager JB, Nair VS, Haber DA, Sequist LV, Alizadeh AA, Diehn M (2020). Integrating genomic features for non-invasive early lung cancer detection. Nature.

[25] Higgins, J.P.T., Green, S. (eds.): Cochrane Handbook for Systematic Reviews of Interventions Version 5.1.0 [updated March 2011]. The Cochrane Collaboration (2011). www.handbook.cochrane.org. Accessed 2 Dec 2020

[26] Strings attached: CADTH’s database search filters. https://www.cadth.ca/resources/finding-evidence/strings-attached-cadths-database-search-filters. Accessed 9 Sept 2019

[27] Drummond MF, Jefferson TO (1996). Guidelines for authors and peer reviewers of economic submissions to the BMJ. BMJ.

[28] Iyer S, Taylor-Stokes G, Roughley A (2013). Symptom burden and quality of life in advanced non-small cell lung cancer patients in France and Germany. Lung Cancer.

[29] Farkkila N, Torvinen S, Roine RP, Sintonen H, Hanninen J, Taari K, Saarto T (2014). Health-related quality of life among breast, prostate, and colorectal cancer patients with end-stage disease. Qual. Life Res..

[30] Hildebrandt T, Thiel FC, Fasching PA, Graf C, Bani MR, Loehberg CR, Schrauder MG, Jud SM, Hack CC, Beckmann MW, Lux MP (2014). Health utilities in gynecological oncology and mastology in Germany. Anticancer Res..

[31] Reichardt P, Leahy M, Garcia Del Muro X, Ferrari S, Martin J, Gelderblom H, Wang J, Krishna A, Eriksson J, Staddon A, Blay JY (2012). Quality of life and utility in patients with metastatic soft tissue and bone sarcoma: the sarcoma treatment and burden of illness in North America and Europe (SABINE) study. Sarcoma.

[32] Wood R, Mitra D, de Courcy J, Iyer S (2017). Patient-reported pain severity, pain interference and health status in HR+/HER2− advanced/metastatic breast cancer. ESMO Open..

[33] Wang L, Shi JF, Zhu J, Huang HY, Bai YN, Liu GX, Liao XZ, Mao AY, Ren JS, Sun XJ, Guo LW, Fang Y, Zhou Q, Ma HM, Xing XJ, Zhu L, Song BB, Du LB, Mai L, Liu YQ, Ren Y, Lan L, Zhou JY, Qi X, Sun XH, Lou PA, Wu SL, Li N, Zhang K, He J, Dai M (2018). Health-related quality of life and utility scores of patients with breast neoplasms in China: a multicenter cross-sectional survey. Breast.

[34] Lidgren M, Wilking N, Jonsson B, Rehnberg C (2007). Health related quality of life in different states of breast cancer. Qual. Life Res..

[35] Guerra RL, Dos Reis NB, Correa FM, Fernandes MM, Fernandes RRA, Cancela MC, Araujo RM, Crocamo S, Santos M, De Almeida LM (2019). Breast cancer quality of life and health-state utility at a Brazilian reference public cancer center. Expert Rev. Pharmacoecon. Outcomes Res..

[36] Kim SH, Jo MW, Ock M, Lee HJ, Lee JW (2017). Estimation of health state utilities in breast cancer. Patient Prefer. Adherence.

[37] Schleinitz MD, DePalo D, Blume J, Stein M (2006). Can differences in breast cancer utilities explain disparities in breast cancer care?. J. Gen. Intern. Med..

[38] Chie WC, Huang CS, Chen JH, Chang KJ (2000). Utility assessment for different clinical phases of breast cancer in Taiwan. J. Formos. Med. Assoc..

[39] Huang W, Yang J, Liu Y, Liu C, Zhang X, Fu W, Shi L, Liu G (2018). Assessing health-related quality of life of patients with colorectal cancer using EQ-5D-5L: a cross-sectional study in Heilongjiang of China. BMJ Open.

[40] Wong G, Howard K, Chapman J, Pollock C, Chadban S, Salkeld G, Tong A, Williams N, Webster A, Craig JC (2012). How do people with chronic kidney disease value cancer-related quality of life?. Nephrology.

[41] Lee JY, Ock M, Jo MW, Son WS, Lee HJ, Kim SH, Kim HJ, Lee JL (2017). Estimating utility weights and quality-adjusted life year loss for colorectal cancer-related health states in Korea. Sci. Rep..

[42] Kim EJ, Ock M, Kim KP, Jung NH, Lee HJ, Kim SH, Jo MW (2018). Disease severity-based evaluation of utility weights for lung cancer-related health states in Korea. BMC Cancer.

[43] Shen Y, Wu B, Wang X, Zhu J (2018). Health state utilities in patients with advanced non-small-cell lung cancer in China. J. Comp. Eff. Res..

[44] Wolff HB, Alberts L, Kastelijn EA, Lissenberg-Witte BI, Twisk JW, Lagerwaard FJ, Senan S, El Sharouni SY, Schramel FMNH, Coupe VMH (2018). Differences in longitudinal health utility between stereotactic body radiation therapy and surgery in stage I non-small cell lung cancer. J. Thorac. Oncol..

[45] Tramontano AC, Schrag DL, Malin JK, Miller MC, Weeks JC, Swan JS, McMahon PM (2015). Catalog and comparison of societal preferences (utilities) for lung cancer health states: results from the Cancer Care Outcomes Research and Surveillance (CanCORS) study. Med. Decis. Mak..

[46] Endarti D, Riewpaiboon A, Thavorncharoensap M, Praditsitthikorn N, Hutubessy R, Kristina SA (2015). Evaluation of health-related quality of life among patients with cervical cancer in Indonesia. Asian Pac. J. Cancer Prev..

[47] Murasawa H, Konno R, Okubo I, Arakawa I (2014). Evaluation of health-related quality of life for hypothesized medical states associated with cervical cancer. Asian Pac. J. Cancer Prev..

[48] Murasawa H, Sugiyama T, Matsuoka Y, Okabe T, Hino A, Tanaka N, Sugimoto M, Oyama M, Fujimoto K, Horie S, Noto S, Shimozuma K (2019). Health utility and health-related quality of life of Japanese prostate cancer patients according to progression status measured using EQ-5D-5L and FACT-P. Qual. Life Res..

[49] Schwarzinger M, Luchini S, Baillot S, Bec M, Benmahammed L, Even C, Geoffrois L, Huguet F, Vu BL, Levy-Bachelot L, Pointreau Y, Robert C, Teyssier LS, Schernberg A, Temam S (2019). Estimating health state utility from activities of daily living in the French National Hospital Discharge Database: a feasibility study with head and neck cancer. Health Qual. Life Outcomes..

[50] Szabo SM, Dobson RL, Donato BMK, L’Italien G, Hotte SJ, Levy AR (2012). The quality-of-life impact of head and neck cancer: preference values from the canadian general public. Health Outcomes Res. Med..

[51] Wildi SM, Cox MH, Clark LL, Turner R, Hawes RH, Hoffman BJ, Wallace MB (2004). Assessment of health state utilities and quality of life in patients with malignant esophageal Dysphagia. Am. J. Gastroenterol..

[52] Liu Q, Zeng H, Xia R, Chen G, Liu S, Zhang Z, Liu Y, Guo G, Song G, Zhu Y, Wu X, Song B, Liao X, Chen Y, Wei W, Chen W, Zhuang G (2018). Health-related quality of life of esophageal cancer patients in daily life after treatment: a multicenter cross-sectional study in China. Cancer Med..

[53] Ruhl, J.L., Callaghan, C., Harlbut, A., Ries, L.A.G., Adamo, P., Dickie, L., Schussler, N.: Summary stage 2018: codes and coding instructions. https://seer.cancer.gov/tools/ssm/2018-Summary-Stage-Manual.pdf (2020). Accessed 22 Dec 2020

[54] Tromme I, Devleesschauwer B, Beutels P, Richez P, Leroy A, Baurain JF, Cornelis F, Bertrand C, Legrand N, Degueldre J, Thomas L, Legrand C, Lambert J, Haagsma J, Speybroeck N (2014). Health-related quality of life in patients with melanoma expressed as utilities and disability weights. Br. J. Dermatol..

[55] Kokkonen K, Tasmuth T, Lehto JT, Kautiainen H, Elme A, Jääskeläinen A-S, Saarto T (2019). Cancer Patients’ Symptom Burden and Health-related Quality of Life (HRQoL) at tertiary cancer center from 2006 to 2013: a cross-sectional study. Anticancer Res..

[56] Meregaglia M, Cairns J (2017). A systematic literature review of health state utility values in head and neck cancer. Health Qual. Life Outcomes..

[57] Jeong, K., Cairns, J.: Systematic review of health state utility values for economic evaluation of colorectal cancer. https://pubmed.ncbi.nlm.nih.gov/27541298/. Accessed 22 Dec 2020 10.1186/s13561-016-0115-5 PMC4991979 27541298

[58] Al-Dakkak I, Borrill J, Murphy E, Posnett J, Zhang Y (2014). A systematic review of health state utility values for advanced ovarian cancer. Value Health..

[59] Siegel RL, Miller KD, Jemal A (2020). Cancer statistics, 2020. CA Cancer J. Clin..

[60] Crosby D, Lyons N, Greenwood E, Harrison S, Hiom S, Moffat J, Quallo T, Samuel E, Walker I (2020). A roadmap for the early detection and diagnosis of cancer. Lancet Oncol..

[61] Arnold D, Girling A, Stevens A, Lilford R (2009). Comparison of direct and indirect methods of estimating health state utilities for resource allocation: review and empirical analysis. BMJ.

[62] NCCN Clinical Practice Guidelines in Oncology (NCCN Guidelines).: Non-Small Cell Lung Cancer (Version 1.2021). https://www.nccn.org/professionals/physician_gls/pdf/nscl.pdf. Accessed 22 Dec 2020

