# Supplementary material for: Health state utility values by cancer stage: a systematic literature review
Source: Eur J Health Econ. 2021 Jun 14;22(8):1275–88. doi: 10.1007/s10198-021-01335-8 (PMC8526485; doi:10.1007/s10198-021-01335-8)
Supplement: Supplementary file 1 — Supplementary file1 (DOCX 20 KB) [file 10198_2021_1335_MOESM1_ESM.docx]

Health state utility values by cancer stage: A systematic literature review

*The European Journal of Health Economics*

Mir-Masoud Pourrahmat, Ashley Kim, Anuraag R. Kansal, Marg Hux, Divya Pushkarna, Mir Sohail Fazeli, Karen C. Chung

Corresponding Author:

Ashley Kim, PharmD, MS

GRAIL, Inc, Menlo Park, California

Email: akim@grailbio.com

**Online Resource 1: PICOS Eligibility Criteria.**

| **PICO item** | **Criteria** | |
| --- | --- | --- |
|  | **Inclusion criteria** | **Exclusion criteria** |
| **Population** | Cancer patients or community members (general population or healthcare experts) valuing scenario descriptions of cancer health states/stages | - Studies with:   - Children or adolescents   - Patients with cancer relapse or precancerous conditions |
| **Interventions** | Any or none | N/A |
| **Comparators** | Any or none | N/A |
| **Outcomes** | Utility estimates, by cancer stage for any cancer type | N/A |
| **Study design** | Studies that developed and reported health utility values for a whole stage of cancer, including:   - Clinical Trials - Observational studies - Surveys and data collection studies (e.g., time-trade off studies) | - Case reports - Case series - Pre-clinical studies - Cost-effectiveness analyses - Cost-utility analyses - Cost-benefit analyses - Cost-minimization analyses - Cost-consequence analyses |
| **Additional criteria (limits)** | | |
| **Timing** | - Any | - N/A |
| **Setting** | - Any | - N/A |
| **Language** | - English only | - Non-English |
| **Publication date limit** | - Last 20 years (2000 – 2019) | - Pre-2000 |
| **Relevant data sources in scope** | | |
| **Published Literature: Main databases** | - Embase (via OvidSP) - MEDLINE^®^ (via OvidSP) - EconLit (via EBSCOhost) | |
| **Grey literature** | - International Society for Pharmacoeconomic and Outcomes Research 2017–2019 (including Europe) - International Conference on Health Economics 2017-2019 - International Society for Quality of Life Research 2017-2019 | |
| N/A, not applicable; PICOS, Population, Intervention, Comparison, Outcomes and Study | | |
