# Supplementary material for: Health state utility values by cancer stage: a systematic literature review
Source: Eur J Health Econ. 2021 Jun 14;22(8):1275–88. doi: 10.1007/s10198-021-01335-8 (PMC8526485; doi:10.1007/s10198-021-01335-8)
Supplement: Supplementary file 2 — Supplementary file2 (DOCX 27 KB) [file 10198_2021_1335_MOESM2_ESM.docx]

Health state utility values by cancer stage: A systematic literature review

*The European Journal of Health Economics*

Mir-Masoud Pourrahmat, Ashley Kim, Anuraag R. Kansal, Marg Hux, Divya Pushkarna, Mir Sohail Fazeli, Karen C. Chung

Corresponding Author:

Ashley Kim, PharmD, MS

GRAIL, Inc, Menlo Park, California

Email: akim@grailbio.com

**Online Resource 2: Search Strategies**

| **Embase 1974 to 2019 September 06 Search executed: September 09, 2019** | | |
| --- | --- | --- |
| **#** | **String** | **Hits** |
| 1 | exp malignant neoplasm/ | 3243579 |
| 2 | (cancer* or carcinoma* or tumo?r* or neoplasm*).ti,ab,kw. | 3879618 |
| 3 | or/1-2 | 4671090 |
| 4 | (sf6 or sf 6 or short form 6 or shortform 6 or sf six or sfsix or shortform six or short form six or shortform6 or short form6).ti,ab,kw. | 2253 |
| 5 | (hye or hyes).ti,ab,kw. | 131 |
| 6 | (health* adj2 year* adj2 equivalent*).ti,ab,kw. | 55 |
| 7 | (quality of wellbeing or quality of well being or index of wellbeing or index of well being or qwb).ti,ab,kw. | 691 |
| 8 | (health adj3 (utilit* or status)).ti,ab,kw. | 88951 |
| 9 | (utilit* adj3 (valu* or measur* or health or life or estimat* or elicit* or disease or score* or weight)).ti,ab,kw. | 18251 |
| 10 | disutilit*.ti,ab,kw. | 856 |
| 11 | standard gamble*.ti,ab,kw. | 1090 |
| 12 | (time trade off or time tradeoff).ti,ab,kw. | 1937 |
| 13 | tto.ti,ab,kw. | 1585 |
| 14 | (hui or hui1 or hui2 or hui3).ti,ab,kw. | 2143 |
| 15 | (eq or euroqol or euro qol or eq5d or eq 5d or euroqual or euro qual).ti,ab,kw. | 24622 |
| 16 | or/4-15 | 125597 |
| 17 | 3 and 16 | 15308 |
| 18 | (book or chapter or conference abstract or conference review or editorial or erratum or letter or note or review or short survey or tombstone).pt. | 9142709 |
| 19 | (exp animal/ or nonhuman/) not exp human/ | 6201152 |
| 20 | or/18-19 | 14605984 |
| 21 | 17 not 20 | 7821 |
| 22 | limit 21 to english | 7300 |
| 23 | limit 22 to yr="1999 -Current" | 6592 |

| **Database: Ovid MEDLINE(R) and Epub Ahead of Print, In-Process & Other Non-Indexed Citations, Daily and Versions(R) 1946 to September 06, 2019**  **Search executed: September 09, 2019** | | |
| --- | --- | --- |
| **#** | **String** | **Hits** |
| 1 | exp Neoplasms/ | 3212932 |
| 2 | (cancer* or carcinoma* or tumo?r* or neoplasm*).ti,ab,kw. | 2929460 |
| 3 | or/1-2 | 4034793 |
| 4 | (sf6 or sf 6 or short form 6 or shortform 6 or sf six or sfsix or shortform six or short form six or shortform6 or short form6).ti,ab,kf. | 2028 |
| 5 | (hye or hyes).ti,ab,kf. | 67 |
| 6 | (health* adj2 year* adj2 equivalent*).ti,ab,kf. | 48 |
| 7 | (quality of wellbeing or quality of well being or index of wellbeing or index of well being or qwb).ti,ab,kf. | 552 |
| 8 | (health adj3 (utilit* or status)).ti,ab,kf. | 67807 |
| 9 | (utilit* adj3 (valu* or measur* or health or life or estimat* or elicit* or disease or score* or weight)).ti,ab,kf. | 11296 |
| 10 | disutilit*.ti,ab,kf. | 435 |
| 11 | standard gamble*.ti,ab,kf. | 834 |
| 12 | (time trade off or time tradeoff).ti,ab,kf. | 1373 |
| 13 | tto.ti,ab,kf. | 1004 |
| 14 | (hui or hui1 or hui2 or hui3).ti,ab,kf. | 1416 |
| 15 | (eq or euroqol or euro qol or eq5d or eq 5d or euroqual or euro qual).ti,ab,kf. | 14529 |
| 16 | or/4-15 | 91411 |
| 17 | 3 and 16 | 8884 |
| 18 | (comment or editorial or meta-analysis or practice-guideline or review or letter or journal correspondence or posters or News or Newspaper article or meeting abstracts or lectures or interview or historical article or handbooks or guidelines or guidebooks or essays or editorial or database or clinical conference or catalogs).pt. | 4896383 |
| 19 | (exp animal/ or nonhuman/) not exp human/ | 4615945 |
| 20 | or/18-19 | 9282586 |
| 21 | 17 not 20 | 7572 |
| 22 | limit 21 to english | 7157 |
| 23 | limit 22 to yr="1999 -Current" | 6485 |
| 24 | Economics/ | 27069 |
| 25 | exp "Costs and Cost Analysis"/ | 227650 |
| 26 | Economics, Nursing/ | 3990 |
| 27 | Economics, Medical/ | 9031 |
| 28 | Economics, Pharmaceutical/ | 2886 |
| 29 | exp Economics, Hospital/ | 23812 |
| 30 | Economics, Dental/ | 1907 |
| 31 | exp "Fees and Charges"/ | 29852 |
| 32 | exp Budgets/ | 13552 |
| 33 | budget*.ti,ab,kf. | 28031 |
| 34 | (economic* or cost or costs or costly or costing or price or prices or pricing or pharmacoeconomic* or pharmaco-economic* or expenditure or expenditures or expense or expenses or financial or finance or finances or financed).ti,kf. | 217032 |
| 35 | (economic* or cost or costs or costly or costing or price or prices or pricing or pharmacoeconomic* or pharmaco-economic* or expenditure or expenditures or expense or expenses or financial or finance or finances or financed).ab. /freq=2 | 269769 |
| 36 | (cost* adj2 (effective* or utilit* or benefit* or minimi* or analy* or outcome or outcomes)).ab,kf. | 151092 |
| 37 | (value adj2 (money or monetary)).ti,ab,kf. | 2233 |
| 38 | exp models, economic/ | 14328 |
| 39 | economic model*.ab,kf. | 3099 |
| 40 | markov chains/ | 13630 |
| 41 | markov.ti,ab,kf. | 20937 |
| 42 | monte carlo method/ | 27112 |
| 43 | monte carlo.ti,ab,kf. | 46542 |
| 44 | exp Decision Theory/ | 11573 |
| 45 | (decision* adj2 (tree* or analy* or model*)).ti,ab,kf. | 21838 |
| 46 | or/24-45 | 691834 |
| 47 | 23 not 46 | 5322 |

| **Database: EconLit**  **Search executed: September 09, 2019** | | |
| --- | --- | --- |
| **#** | **String** | **Hits** |
| S1 | (cancer* or carcinoma* or tumo?r* or neoplasm*)  Limiters - Published  Date: 19990101-20191231  Expanders - Apply equivalent subjects  Narrow by Language: -english  Search modes -Boolean/Phrase | 1486 |

| **Database: ISPOR Presentations Database**  **Search executed: October 28, 2019** | |
| --- | --- |
| **String** | **Hits** |
| Disease/Disorder: Oncology  Topic: Patient-Centered Research  Subtopic: Health State Utilities | 1486 |
| Conference: 2019-11, ISPOR Europe 2019, Copenhagen, Denmark | 12 |
| Conference: 2019-09, ISPOR Latin American 2019, Bogota, Colombia | 0 |
| Conference: 2019-05, ISPOR 2019, New Orleans, LA, UA | 1 |
| Conference: 2018-11, ISPOR Europe 2018, Barcelona, Spain | 15 |
| Conference: 2018-09, ISPOR Asia Pacific 2018, Tokyo, Japan | 0 |
| Conference: 2018-05, ISPOR 2018, Baltimore, MD, USA | 5 |
| Conference: 2017-11, ISPOR Europe 2017, Glasgow, Scotland | 17 |
| Conference: 2017-09, ISPOR Latin American 2017, Sao Paulo, Brazil | 0 |
| Conference: 2017-05, ISPOR 2017, Boston, MA, UA | 6 |
| Total | 56 |
