# Supplementary material for: Health state utility values by cancer stage: a systematic literature review
Source: Eur J Health Econ. 2021 Jun 14;22(8):1275–88. doi: 10.1007/s10198-021-01335-8 (PMC8526485; doi:10.1007/s10198-021-01335-8)
Supplement: Supplementary file 3 — Supplementary file3 (DOCX 53 KB) [file 10198_2021_1335_MOESM3_ESM.docx]

Health state utility values by cancer stage: A systematic literature review

*The European Journal of Health Economics*

Mir-Masoud Pourrahmat, Ashley Kim, Anuraag R. Kansal, Marg Hux, Divya Pushkarna, Mir Sohail Fazeli, Karen C. Chung

Corresponding Author:

Ashley Kim, PharmD, MS

GRAIL, Inc, Menlo Park, California

Email: akim@grailbio.com

Online Resource 3: Summary of Patient and Study Characteristics

| **Author (Year)** | **Study Country** | **Study Design** | **Study Enrollment Period** | **Cancer Type** | **Reported Cancer Stage/Health State** | **N** | **Mean (SD) age, y** | **% Male** | **Mean Disease Duration** |  |
| --- | --- | --- | --- | --- | --- | --- | --- | --- | --- | --- |
| Chie (2000) [38] | Taiwan | Cross sectional | Jul-Sept 1997 | Breast cancer | Early | 21 | NR | 16 | NR |  |
|  |  |  |  |  | Late stage | 21 | NR | 16 | NR |  |
|  |  |  |  |  | Terminal | 21 | NR | 16 | NR |  |
|  |  |  |  |  | Terminal hospice | 21 | NR | 16 | NR |  |
| Endarti (2015) [46] | Indonesia | Cross sectional | Jun-Dec 2013 | Cervical cancer | Stage I | 12 | NR | NR | NR |  |
|  |  |  |  |  | Stage II | 44 | NR | NR | NR |  |
|  |  |  |  |  | Stage III | 27 | NR | NR | NR |  |
|  |  |  |  |  | Stage IV | 4 | NR | NR | NR |  |
| Farkkila (2014) [29] | Finland | Cross sectional | Sep 2009‑Apr 2011 | Breast cancer | End stage | 27 | 66 | 0 | 6.6 y |  |
|  |  |  |  | Prostate cancer | End stage | 30 | 75 | 100 | 7.8 y |  |
|  |  |  |  | Colorectal cancer | End stage | 57 | 69 | 67 | 2.9 y |  |
| Guerra (2019) [35] | Brazil | Prospective | Mar-May 2017 | Breast cancer | Stages 0–II | 104 | NR | 0 | NR |  |
|  |  |  |  |  | Stages III–IV | 90 | NR | 0 | NR |  |
| Hildebrandt (2014) [30] | Germany | Cross sectional | May-Dec 2009 | Breast cancer | NR | 442 | NR | NR | NR |  |
|  |  |  |  | Ovarian cancer | NR | 37 | NR | 0 | NR |  |
|  |  |  |  | Endometrial cancer | NR | 20 | NR | NR | NR |  |
|  |  |  |  | Cervical cancer | NR | 16 | NR | 0 | NR |  |
| Huang (2018) [39] | China | Cross sectional | Dec 2016-Apr 2017 | Colorectal cancer | Stage I | 40 | NR | NR | NR |  |
|  |  |  |  |  | Stage II | 111 | NR | NR | NR |  |
|  |  |  |  |  | Stage III | 114 | NR | NR | NR |  |
|  |  |  |  |  | Stage IV | 35 | NR | NR | NR |  |
| Iyer (2013) [28] | France, Germany | Cross sectional | Jul-Oct 2010 | NSCLC | Stage IIIB/IV | 837 | 63 | 67 | 232.4 d |  |
| Kim (2018) [42] | South Korea | Cross sectional | NR | Lung cancer | Stage I | NR | NR | NR | NR |  |
|  |  |  |  |  | Stage II | NR | NR | NR | NR |  |
|  |  |  |  |  | Stage IIIa | NR | NR | NR | NR |  |
|  |  |  |  |  | Stage IIIB | NR | NR | NR | NR |  |
|  |  |  |  |  | Stage IV | NR | NR | NR | NR |  |
| Kim (2017) [36] | South Korea | Cross sectional | Mar-Apr 2016 | Breast cancer | Invasive, with mastectomy or breast-conserving surgery, radiation therapy, and/or chemotherapy (I, II^a^) | 509 | NR | NR | NR |  |
|  |  |  |  |  | Locally advanced, with radical mastectomy and radiation therapy (IIIA, IIIB^a^) | 509 | NR | NR | NR |  |
|  |  |  |  |  | Inoperable, locally advanced (IIIC^a^) | 509 | NR | NR | NR |  |
|  |  |  |  |  | Metastatic (IV^a^) | 509 | NR | NR | NR |  |
| Lee (2017) [41] | South Korea | Cross sectional | NR | Colorectal cancer | HS 2: Colon cancer requiring colon resection | 402 | NR | NR | NR |  |
|  |  |  |  |  | HS 3: Rectal cancer requiring rectal resection | 396 | NR | NR | NR |  |
|  |  |  |  |  | HS 4: Colon cancer requiring colon resection and systemic chemotherapy | 407 | NR | NR | NR |  |
|  |  |  |  |  | HS 5: Rectal cancer requiring rectal resection and chemoradiation therapy | 422 | NR | NR | NR |  |
|  |  |  |  |  | HS 6: Rectal cancer requiring rectal resection, stoma formation, and chemoradiation therapy | 392 | NR | NR | NR |  |
|  |  |  |  |  | HS 7: Metastatic colon cancer | 381 | NR | NR | NR |  |
| Lidgren (2007) [34] | Sweden | Cross sectional | Apr-May 2005 | Breast cancer | HS P: First year after primary breast cancer | 72 | NR | 0 | NR |  |
|  |  |  |  |  | HS R: First year after recurrence | 21 | NR | 0 | NR |  |
|  |  |  |  |  | HS M: Metastatic | 65 | NR | 0 | NR |  |
| Liu (2018) [52] | China | Cross sectional | Oct 1, 2016 to Mar 31, 2017 | Esophageal cancer | Stage I | 313 | NR | 73.5 | NR |  |
|  |  |  |  |  | Stage II | 381 | NR | 76.4 | NR |  |
|  |  |  |  |  | Stage III | 288 | NR | 76.7 | NR |  |
|  |  |  |  |  | Stage IV | 204 | NR | 74 | NR |  |
| Matza (2014) [16] | UK, Canada | Cross sectional | UK: Jul 2010 Montreal: Sept 2010  Toronto: Dec 2010 | Bone metastasis | UK |  |  |  |  |  |
|  |  |  |  |  | A: Basic HS (no SRE) | 126 | NR | NR | NR |  |
|  |  |  |  |  | B: Basic HS + spinal cord compression without paralysis | 126 | NR | NR | NR |  |
|  |  |  |  |  | C: Basic HS + spinal cord compression with paralysis | 126 | NR | NR | NR |  |
|  |  |  |  |  | D: Basic HS + leg fracture | 126 | NR | NR | NR |  |
|  |  |  |  |  | E: Basic HS + rib fracture | 126 | NR | NR | NR |  |
|  |  |  |  |  | F: Basic HS + arm fracture | 126 | NR | NR | NR |  |
|  |  |  |  |  | G: Basic HS + radiation treatment (5 appointments x 2 weeks) | 126 | NR | NR | NR |  |
|  |  |  |  |  | H: basic HS + radiation treatment (2 appointments) | 126 | NR | NR | NR |  |
|  |  |  |  |  | I: basic HS + surgery to stabilize bone | 126 | NR | NR | NR |  |
|  |  |  |  |  | Canada |  |  |  |  |  |
|  |  |  |  |  | A: Basic HS (no SRE) | 61 | NR | NR | NR |  |
|  |  |  |  |  | B: Basic HS + spinal cord compression without paralysis | 61 | NR | NR | NR |  |
|  |  |  |  |  | C: Basic HS + spinal cord compression with paralysis | 61 | NR | NR | NR |  |
|  |  |  |  |  | D: Basic HS + leg fracture | 61 | NR | NR | NR |  |
|  |  |  |  |  | E: Basic HS + rib fracture | 61 | NR | NR | NR |  |
|  |  |  |  |  | F: Basic HS + arm fracture | 61 | NR | NR | NR |  |
|  |  |  |  |  | G: Basic HS + radiation treatment (5 appointments x 2 weeks) | 61 | NR | NR | NR |  |
|  |  |  |  |  | H: Basic HS + radiation treatment (2 appointments) | 61 | NR | NR | NR |  |
|  |  |  |  |  | I: Basic HS + surgery to stabilize bone | 61 | NR | NR | NR |  |
| Murasawa (2014) [47] | Japan | Cross sectional | Oct 2013 | Cervical cancer | Stage IA1 | 27 | NR | 0 | NR |  |
|  |  |  |  |  | Stage IA2 | 24 | NR | 0 | NR |  |
|  |  |  |  |  | Stage IB1 | 21 | NR | 0 | NR |  |
|  |  |  |  |  | Stage IB2 | 26 | NR | 0 | NR |  |
|  |  |  |  |  | Stage IIA | 19 | NR | 0 | NR |  |
|  |  |  |  |  | Stage IIB | 24 | NR | 0 | NR |  |
|  |  |  |  |  | Stage III | 24 | NR | 0 | NR |  |
|  |  |  |  |  | Stage IV | 23 | NR | 0 | NR |  |
| Murasawa (2019) [48] | Japan | Cross sectional | Feb-Dec 2017 | Prostate cancer | Localized | 275 | 73.2 | 100 | NR |  |
|  |  |  |  |  | Localized progressive | 40 | 73 | 100 | NR |  |
|  |  |  |  |  | Distant metastatic | 27 | 73.7 | 100 | NR |  |
|  |  |  |  |  | Distant metastatic castration-resistant prostate cancer | 38 | 73.7 | 100 | NR |  |
| Reichardt (2012) [31] | Canada, USA, Germany, France, Italy, The Netherlands, Spain, UK, and Sweden | Cross sectional | Dec 2009-Mar 2011 | Sarcoma | HS 1: 1st-line chemotherapy, pre-progressive disease | 17 | NR | NR | NR |  |
|  |  |  |  |  | HS 2: 2nd-line chemotherapy, pre-progressive disease | 22 | NR | NR | NR |  |
|  |  |  |  |  | HS 3: 3rd-line or higher chemotherapy, pre-progressive disease | 12 | NR | NR | NR |  |
|  |  |  |  |  | HS 4: Progressive disease (on or off chemotherapy) | 28 | NR | NR | NR |  |
|  |  |  |  |  | HS 5: After chemotherapy, pre-progressive disease | 35 | NR | NR | NR |  |
| Schleinitz (2006) [37] | USA | Cross sectional | Aug 2003-Jun 2004 | Breast cancer | Stage I | NR | NR | 0 | NR |  |
|  |  |  |  |  | Stage II | NR | NR | 0 | NR |  |
|  |  |  |  |  | Stage III | NR | NR | 0 | NR |  |
|  |  |  |  |  | Stage IV- | NR | NR | 0 | NR |  |
|  |  |  |  |  | Stage IV+ | NR | NR | 0 | NR |  |
| Schwarzinger (2019) [49] | France | Cross sectional | 2008-2013 | Head and neck cancer | Early stage, initial treatment | 87,965 | NR | 0 | NR |  |
|  |  |  |  |  | Locally advanced, initial treatment | 181,094 | NR | 0 | NR |  |
|  |  |  |  |  | Distant metastasis, initial treatment | 33,573 | NR | 0 | NR |  |
| Shen (2018) [43] | China | Cross sectional | Jan-Jun 2016 | NSCLC | Stage III | 55 | NR | NR | NR |  |
|  |  |  |  |  | Stage IV | 173 | NR | NR | NR |  |
| Szabo (2012) [50] | Canada | Cross sectional | Feb-Apr 2009 | Head and neck cancer | Locoregional, laryngeal | 101 | 47.5 (85) | 47.5 | NR |  |
|  |  |  |  |  | Locoregional, nonlaryngeal | 101 | 47.5 (85) | 47.5 | NR |  |
|  |  |  |  |  | Metastatic, nonlaryngeal | 101 | 47.5 (85) | 47.5 | NR |  |
|  |  |  |  |  | Metastatic, laryngeal | 101 | 47.5 (85) | 47.5 | NR |  |
| Tramontano (2015) [45] | USA | Cross sectional | 2003-2005 | Lung cancer | Stage I | 750 | NR | NR | NR |  |
|  |  |  |  |  | Stage II | 232 | NR | NR | NR |  |
|  |  |  |  |  | Stage III | 649 | NR | NR | NR |  |
|  |  |  |  |  | Stage IV | 628 | NR | NR | NR |  |
| Tromme (2014) [54] | Belgium | Cross sectional | July 1, 2012-Dec 31, 2012  Extra inclusion period for patients in treatment only: Jan-May 2013 | Melanoma | Stage IB/II-T | 33 | 54.5 (80) | NR | NR |  |
|  |  |  |  |  | Stage IB/II-R | 76 | 53.2 (80) | NR | NR |  |
|  |  |  |  |  | Stage III-T | 15 | 55.9 (83) | NR | NR |  |
|  |  |  |  |  | Stage III-R | 50 | 53.3 (86) | NR | NR |  |
|  |  |  |  |  | Stage IV-T | 41 | 61.4 (84) | NR | NR |  |
|  |  |  |  |  | Stage IV-R | 14 | 64.8 (84) | NR | NR |  |
| Wang (2018) [33] | China | Cross sectional | Sept 2013-Dec 2014 | Breast cancer | Stage I | 498 | 49.5 | 0 | NR |  |
|  |  |  |  |  | Stage II | 1,234 | 49.8 | 0 | NR |  |
|  |  |  |  |  | Stage III | 556 | 50.2 | 0 | NR |  |
|  |  |  |  |  | Stage IV | 224 | 50.7 | 0 | NR |  |
| Wildi (2004) [51] | USA | Cross sectional | NR | Esophageal cancer | Stage I | 11 | NR | NR | NR |  |
|  |  |  |  |  | Stage II | 24 | NR | NR | NR |  |
|  |  |  |  |  | Stage III | 12 | NR | NR | NR |  |
| Wolff (2018) [44] | The Netherlands | Prospective | Cohort 1: Mar 2013-Jan 2016 Cohort 2: Apr 2003-Nov 2008 | NSCLC | Stage IA | 261 | 66.7 | 63.6 | NR |  |
|  |  |  |  |  | Stage IB | 41 | 74.3 | 58.5 | NR |  |
| Wong (2012) [40] | Australia | Cross sectional | Sept 2009-Jun 2010 | Colorectal cancer | CKD stage 3-5 | 53 | NR | 52.8 | NR |  |
|  |  |  |  |  | Dialysis | 89 | NR | 60.7 | NR |  |
|  |  |  |  |  | Transplant | 79 | NR | 63.3 | NR |  |
| Wood (2017) [32] | USA, France, Germany, Italy, Spain, UK | Cross sectional | Feb-May 2015 | Breast cancer | Stage IV | 611 | NR | 0 | NR |  |
| CIS, carcinoma in situ; CKD, chronic kidney disease; HS: health state; NR, not reported; NSCLC, non-small-cell lung cancer; SRE, skeletal-related events; UK, United Kingdom; USA, United States of America ^a^As defined by the 7th edition of the American Joint Committee on Cancer staging manual | | | | | | | | | | |
