# Supplementary material for: Health state utility values by cancer stage: a systematic literature review
Source: Eur J Health Econ. 2021 Jun 14;22(8):1275–88. doi: 10.1007/s10198-021-01335-8 (PMC8526485; doi:10.1007/s10198-021-01335-8)
Supplement: Supplementary file 5 — Supplementary file5 (DOCX 26 KB) [file 10198_2021_1335_MOESM5_ESM.docx]

Health state utility values by cancer stage: A systematic literature review

*The European Journal of Health Economics*

Mir-Masoud Pourrahmat, Ashley Kim, Anuraag R. Kansal, Marg Hux, Divya Pushkarna, Mir Sohail Fazeli, Karen C. Chung

Corresponding Author:

Ashley Kim, PharmD, MS

GRAIL, Inc, Menlo Park, California

Email: akim@grailbio.com

Online Resource 5: Modified NICE Single Technology Appraisal Results

| **Author Year [Reference]** | **Study Design** | | **Data Collection** | | | | **Other sources of bias** | | |
| --- | --- | --- | --- | --- | --- | --- | --- | --- | --- |
|  | **Was the research question stated as a utility development in cancer states?** | **Are utility values reported for whole stages of cancer; overall or by specific types?** | **Were the methods used to value health states and other benefits stated?** | **Were the details of the subjects from whom valuations were obtained given?** | **Were details of any model used for analysis of utility values given?** | **Was there a justification for the choice of model used and the key parameters on which it was based?** | **Was the answer to the study question given?** | **Did conclusions follow from the data reported?** | **Were conclusions accompanied by the appropriate caveats?** |
| Chie 2000 [38] | Yes | Yes | Yes | No | No | N/A | Yes | Not clear | Yes |
| Endarti 2015 [46] | No | Yes | Yes | Yes | No | N/A | Yes | Yes | Yes |
| Farkkila 2014 [29] | Yes | Yes | Yes | Yes | Yes | No | Yes | Yes | Yes |
| Guerra 2019 [35] | Yes | Yes | Yes | Yes | No | N/A | Yes | Yes | Yes |
| Hildebrandt 2014 [30] | Yes | Yes | Yes | Yes | No | N/A | Yes | Yes | Yes |
| Huang 2018 [39] | No | Yes | Yes | Yes | Yes | No | Yes | Yes | Yes |
| Iyer 2013 [28] | No | Yes | Yes | Yes | No | N/A | Yes | Yes | Yes |
| Kim 2018 [42] | Yes | Yes | Yes | Yes | Yes | No | Yes | Yes | Yes |
| Kim 2017 [36] | Yes | Yes | Yes | Yes | No | N/A | Yes | Yes | Yes |
| Lee 2017 [41] | Yes | Yes | Yes | Yes | No | N/A | Yes | Yes | Yes |
| Lidgren 2007 [34] | Yes | Yes | Yes | Yes | Yes | Yes | Yes | Yes | Yes |
| Liu 2018 [52] | No | Yes | Yes | Yes | Yes | Yes | Yes | Yes | Yes |
| Matza 2014 [16] | Yes | Yes | Yes | Yes | No | N/A | Yes | Yes | Yes |
| Murasawa 2014 [47] | Yes | Yes | Yes | Yes | Yes | No | Yes | Yes | Yes |
| Murasawa 2019 [48] | Yes | Yes | Yes | Yes | Yes | No | Yes | Yes | Yes |
| Reichardt 2012 [31] | No | Yes | Yes | Yes | No | N/A | Yes | Yes | Yes |
| Schleinitz 2006 [37] | Yes | Yes | Yes | Yes | Yes | Yes | Yes | Yes | Yes |
| Schwarzinger 2019 [49] | Yes | Yes | Yes | No | No | N/A | Yes | Yes | Yes |
| Shen 2019 [43] | Yes | Yes | Yes | Yes | Yes | No | Yes | Yes | Yes |
| Szabo 2018 [50] | Yes | Yes | Yes | Yes | Yes | Yes | Yes | Yes | Yes |
| Tramontano 2015 [45] | Yes | Yes | Yes | Yes | Yes | No | Yes | Not clear | Yes |
| Tromme 2014 [54] | Yes | Yes | Yes | Yes | No | N/A | Yes | Yes | Yes |
| Wang 2018 [33] | Yes | Yes | Yes | Yes | Yes | Yes | Yes | Yes | Yes |
| Wildi 2004 [51] | Yes | Yes | Yes | Yes | Yes | No | Yes | Yes | No |
| Wolff 2018 [44] | Yes | Yes | Yes | Yes | Yes | No | Yes | Yes | Yes |
| Wong 2012 [40] | Yes | Yes | Yes | Yes | Yes | No | Yes | Yes | Yes |
| Wood 2017 [32] | No | Yes | Yes | Yes | Yes | No | Yes | Yes | Yes |
| N/A, not applicable; NICE, National Institute for Health and Care Excellence | | | | | | | | | |
